# Supplementary material for: Efficacy, safety, and immunogenicity of Lassa fever vaccines: A living systematic review and landscape analysis of vaccine candidates
Source: PLoS One. 2025 Dec 17;20(12):e0338128. doi: 10.1371/journal.pone.0338128 (PMC12711090; doi:10.1371/journal.pone.0338128)
Supplement: S1 File — (DOCX) [file pone.0338128.s001.docx]

### **S1 File. Search strategy**

| Search | Query |
| --- | --- |
| #12 | #6 AND #11 |
| #11 | #7 OR #8 OR #9 OR #10 |
| #10 | Immun*[tiab] |
| #9 | Immunogenicity, Vaccine[Mesh] |
| #8 | Vaccin*[tiab] |
| #7 | Immunization[Mesh] |
| #6 | #1 OR #2 OR #3 OR #4 OR #5 |
| #5 | LASV[tiab] |
| #4 | Old-World Arenavir*[tiab] |
| #3 | Lassa[tiab] |
| #2 | Lassa Virus[Mesh] |
| #1 | Lassa Fever[Mesh] |

### **S1 Table 1. Excluded studies and reason for exclusion**

| Author, year | Title | Reason for exclusion |
| --- | --- | --- |
| Baize, S (2020) | The measles-vectored lassa vaccine MV-LASV is safe and immunogenic-interim results from a first in man phase 1 clinical trial | Duplicate |
| Lukashevich, I (2008) | Safety and Efficacy of ML29 Reassortant Lassa Fever Vaccine in Non-Human Primates | Duplicate |
| Morrison, H (1989) | PROTECTION OF GUINEA-PIGS FROM LASSA FEVER BY VACCINIA VIRUS RECOMBINANTS EXPRESSING THE NUCLEOPROTEIN OR THE ENVELOPE GLYCOPROTEINS OF LASSA VIRUS | Duplicate |
| Carey, D (2025) | A safe and efficacious Lassa virus live-attenuated vaccine candidate based on rearrangement of the intergenic region and codon-deoptimization of the glycoprotein gene | Duplicate |
| Ignat’ev, G (2002) | [Immunogenic and protective characteristics of recombinant Lassa virus NP protein] | No full text available |
| Ignat’ev, G (1989) | [Characteristics of the immune response in mice immunized with the inactivated Lassa virus] | No full text available |
| Krasnianskia, V (1993) | [A trial to produce an inactivated Lassa fever vaccine] | No full text available |
| NCT, 2023 | A Lassa Fever Vaccine Trial in Adults and Children Residing in West Africa | No results posted |
| PACTR, 2021 | A Phase 1 Randomized, Blinded, Placebo Controlled, Dose-Escalation and Dosing Regimen Selection Study to Evaluate the Safety and Immunogenicity of rVSV-Vectored Lassa Virus Vaccine in Healthy Adults at Multiple Sites in West Africa. | No results posted |
| PACTR, 2022 | A Phase 2 Lassa fever vaccine trial in adults and children residing in West Africa | No results posted |
| NCT (2021) | A Clinical Trial to Evaluate the Safety and Immunogenicity of rVSVâˆ†G-LASV-GPC Vaccine in Adults in Good General Heath | Not results posted |
| PACTR (2019) | Dose-ranging Study: safety, Tolerability and Immunogenicity of INO-4500 in Healthy Volunteers in Ghana | Not results posted |
| Barkar, N (1989) | [Lassa and Mozambique viruses: cross protection in experiments on mice and action of immunosuppressants on experimental infections] | Wrong intervention |
| Bozzo, J (2017) | Use of human immunoglobulins as an anti-infective treatment: the experience so far and their possible re-emerging role. | Wrong intervention |
| No name (1987) | GENETICALLY ENGINEERED VACCINE FOR LASSA FEVER | Wrong intervention |
| Auperin, D (1988) | Construction of a recombinant vaccinia virus expressing the Lassa virus glycoprotein gene and protection of guinea pigs from a lethal Lassa virus infection | Wrong intervention |
| Cashman, K (2022) | Lassa antiviral LHF-535 protects guinea pigs from lethal challenge | Wrong intervention |
| Cheng, BY (2017) | Development of live-attenuated arenavirus vaccines based on codon deoptimization of the viral glycoprotein | Wrong intervention |
| Fishman, J (2014) | VaxCelerate: The use of MTBhsp70-avidin as an adjuvant to rapidly generate self-assembling vaccines with biotinylated, antigen-specific peptides targeting emerging pathogens | Wrong intervention |
| Flaxman, A (2019) | An effective multi-pathogen vaccine targeting filoviruses and an arenavirus | Wrong intervention |
| Gemechu, Y (2023) | A NOVEL SELF-ASSEMBLING VACCINE, VTX-067, TARGETING E6/E7 PROTEINS OF HUMAN PAPILLOMA VIRUS INDUCES T CELL IMMUNE RESPONSES AND INHIBITS HPV E6/E7 EXPRESSING TUMOR GROWTH IN A C57/B6 MOUSE MODEL | Wrong intervention |
| Hasche, D (2019) | Mastomys species as model systems for infectious diseases | Wrong intervention |
| La Posta, V (1993) | Cross-protection against lymphocytic choriomeningitis virus mediated by a CD4+ T-cell clone specific for an envelope glycoprotein epitope of Lassa virus | Wrong intervention |
| Reynard, S (2023) | A MOPEVAC multivalent vaccine induces sterile protection against New World arenaviruses in non-human primates | Wrong intervention |
| Anyebe, V (2023) | LASSA FEVER VACCINE TRIAL PREPAREDNESS: PRELIMINARY FINDINGS OF A TARGETED COMMUNITYBASED EPIDEMIOLOGIC STUDY IN NIGERIA | Wrong outcome |
| Hart, C (1988) | Pregnancy and host resistance | Wrong outcome |
| Ignat’ev, G (1989) | Characteristics of the immune response in mice immunized with inactivated Lassa virus | Wrong outcome |
| Ignat’ev, G (1989) | CHARACTERISTICS OF THE IMMUNE-RESPONSE IN MICE IMMUNIZED WITH INACTIVATED LASSA VIRUS | Wrong outcome |
| Krasnianskia, V (1993) | Experience of preparing inactivated vaccine against Lassa fever | Wrong outcome |
| Lu, K (2021) | U.S.-Japan cooperative medical sciences program: 22nd International Conference on Emerging Infectious Diseases in the Pacific Rim | Wrong outcome |
| Pinschewer, D (2010) | Innate and adaptive immune control of genetically engineered live-attenuated arenavirus vaccine prototypes | Wrong outcome |
| Wilson, B (2025) | INFORMING LASSA FEVER VACCINE TRIAL IMPLEMENTATION THROUGH COMMUNITY ENGAGEMENT | Wrong outcome |
| No name (2018) | EARLY TESTS SHOW PROMISE FOR LASSA, RABIES VACCINE | Wrong study design |
| No name (2022) | LASSA FEVER VACCINE GIVES MONKEYS FAST PROTECTION | Wrong study design |
| Anonymous (2019) | 20th Annual International Meeting of the Institute of Human Virology | Wrong study design |
| Cernuschi, T (2024) | The quest for more effective vaccine markets - Opportunities, challenges, and what has changed with the SARS-CoV-2 pandemic | Wrong study design |
| De Groot, A (2020) | Better Epitope Discovery, Precision Immune Engineering, and Accelerated Vaccine Design Using Immunoinformatics Tools | Wrong study design |
| Devi, S (2022) | New vaccine for Lassa fever | Wrong study design |
| Ewer, K (2017) | Chimpanzee adenoviral vectors as vaccines for outbreak pathogens | Wrong study design |
| Hashizume, M (2024) | An mRNA-LNP-based Lassa virus vaccine induces protective immunity in mice | Wrong study design |
| Ibukun, F (2020) | Inter-Lineage Variation of Lassa Virus Glycoprotein Epitopes: A Challenge to Lassa Virus Vaccine Development | Wrong study design |
| Isaac, A (2002) | PROSPECTS OF LASSA FEVER CANDIDATE VACCINES | Wrong study design |
| Jerath, R (1979) | Recent advances in viral zoonoses | Wrong study design |
| Lukashevich, I (2012) | Advanced vaccine candidates for Lassa fever | Wrong study design |
| Lukashevich, I (2016) | Vaccine platforms to control Lassa fever | Wrong study design |
| Medugu, N (2023) | A review of the recent advances on Lassa fever with special reference to molecular epidemiology and progress in vaccine development | Wrong study design |
| Popova, O (2021) | [Review of candidate vaccines for the prevention of Lassa fever] | Wrong study design |
| Purushotham, J (2019) | Vaccine platforms for the prevention of Lassa fever | Wrong study design |
| Rowaiye, A (2022) | Identifying immunodominant multi-epitopes from the envelope glycoprotein of the Lassa mammarenavirus as vaccine candidate for Lassa fever | Wrong study design |
| Salami, K (2019) | A review of Lassa fever vaccine candidates | Wrong study design |
| Salami, K (2020) | A systematic scorecard-based approach to site assessment in preparation for Lassa fever vaccine clinical trials in affected countries | Wrong study design |
| Warner, B (2018) | Current research for a vaccine against Lassa hemorrhagic fever virus | Wrong study design |
| Zapata, J (2013) | Transcriptome analysis of human peripheral blood mononuclear cells exposed to Lassa virus and to the attenuated Mopeia/Lassa reassortant 29 (ML29), a vaccine candidate | Wrong study design |

### **S1 Table 2. Risk of Bias Assessment for Randomized Controlled Trials**

###
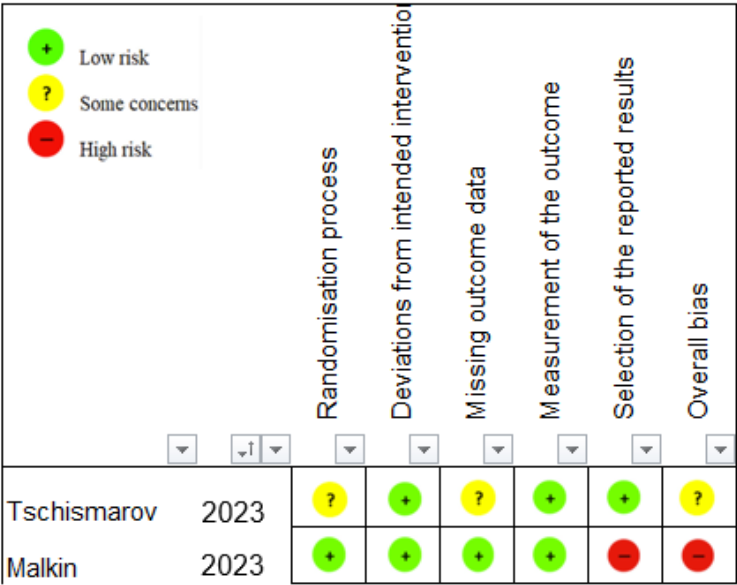


###


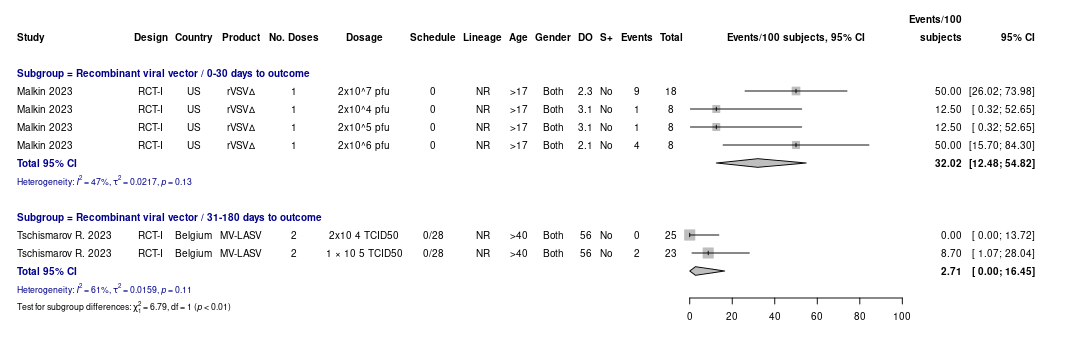


**S1 Fig A. Forest plot of the meta-analysis for arthralgia**

**S1 Fig B. Forest plot of the meta-analysis for Diarrhea**
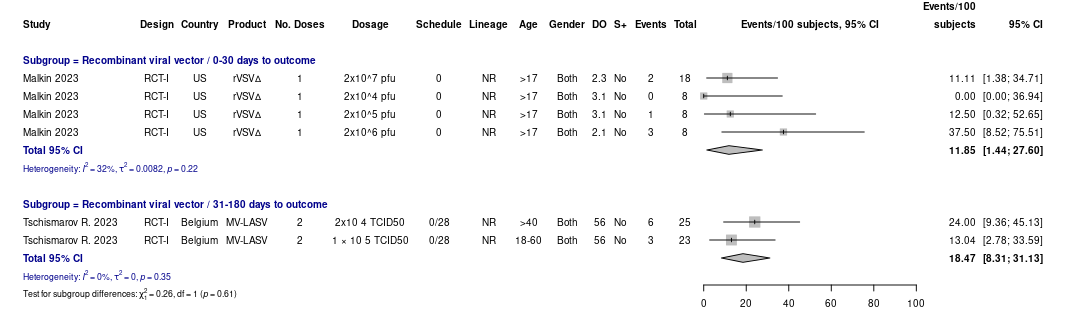


**S1 Fig C. Forest plot of the meta-analysis for Fever**
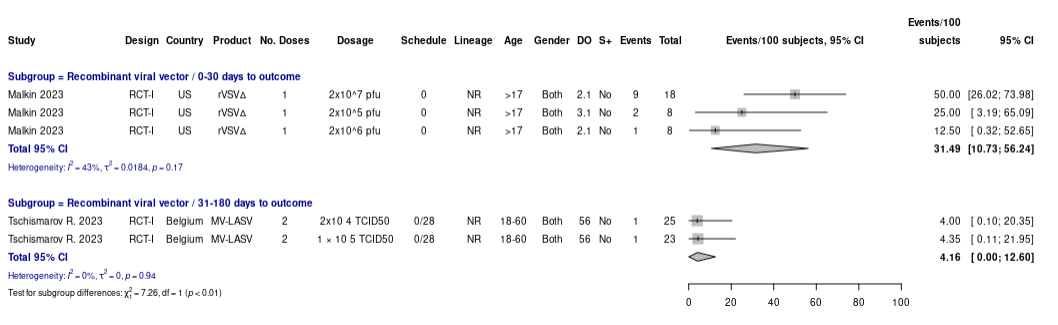


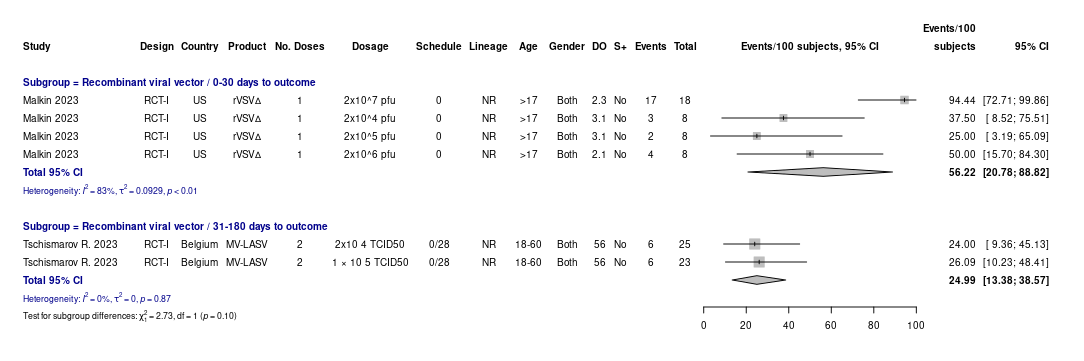


**S1 Fig D. Forest plot of the meta-analysis for Myalgia**

**S1 Fig A-D. Meta-analyses of adverse events following vaccination with Lassa vaccines.** Panels show different adverse events reported in two randomized controlled trials (RCTs) from the United States (rVSVΔ vaccine, single-dose) and Belgium (MV-LASV vaccine, two-dose regimen). Outcomes analyzed include arthralgia (S1 Fig A), diarrhea (S1 Fig B), fever (S1 Fig C), and myalgia (S1 Fig D). Each panel presents subgroup analyses by post-vaccination time intervals (0–30 days and 31–180 days), event rates per 100 subjects with 95% confidence intervals, and heterogeneity statistics (I² and associated p-values)

**S1 Table 3. Preclinical Lassa Fever Vaccine Candidates with Discontinued Status**

| **Platform** | **Vaccine name** | **Author (year)** | **Country** | **Animals** | **Status** | **Immunogenicity endpoint** | **Efficacy endpoint** | **Safety endpoint** |
| --- | --- | --- | --- | --- | --- | --- | --- | --- |
| **DNA** | INO-4500 | Andrade, V (2024) [[36]](https://paperpile.com/c/M9WNpt/RSL3) | United States | Primates | Discontinued | ✓ | ✓ |  |
|  | INO-4500 | Jiang, J (2019) [[38]](https://paperpile.com/c/M9WNpt/b60X) | United States | Rodents and Primates | Discontinued | ✓ | ✓ |  |
|  | INO-4500 | Cashman, K (2017) [[39]](https://paperpile.com/c/M9WNpt/n9bL) | United States | Primates | Discontinued | ✓ | ✓ |  |
|  | INO-4500 | Cashman, K (2017) [[40]](https://paperpile.com/c/M9WNpt/Dt7w) | United States | Rodents | Discontinued | ✓ | ✓ |  |
| **Recombinant** | PS (LASV-GP1) | Galan Navarro, C (2017) [[65]](https://paperpile.com/c/M9WNpt/ybLS) | Switzerland | Rodents | Discontinued | ✓ |  |  |
| **Recombinant Viral vector** | EBS-LASV | Matassov, D (2024) [[62]](https://paperpile.com/c/M9WNpt/zMlK) | United States | Rodents | Discontinued | ✓ |  | ✓ |
| **Recombinant Viral vector (cont.)** | MV-LASV | Mateo, M (2023) [[78]](https://paperpile.com/c/M9WNpt/4NXq) | France | Primates | Discontinued | ✓ | ✓ |  |
|  | MV-LASV | Schrauf, S (2023) [[79]](https://paperpile.com/c/M9WNpt/mHfW) | United States | Primates | Discontinued | ✓ | ✓ |  |
|  | MV-LASV | Mateo, M (2021) [[82]](https://paperpile.com/c/M9WNpt/lJVh) | France | Primates | Discontinued | ✓ | ✓ |  |
|  | MV-LASV | Mateo, M (2019) [[84]](https://paperpile.com/c/M9WNpt/IbNM) | France | Primates | Discontinued | ✓ |  | ✓ |

**S1 Table 4. Status of Lassa Fever Vaccine Candidates in Clinical Development (expanded version)**

| **Platform** | **Candidate** | **Phase** | **Year** | **Location** | **Study design** | **Population** | **N** | **Primary outcome(s)** | **Funding** | **Current status** | **Trial registration** | **Dosage** |
| --- | --- | --- | --- | --- | --- | --- | --- | --- | --- | --- | --- | --- |
| Recombinant viral vector | rVSVΔG-LASV-GPC | 2b | 20222023 | Ghana, Liberia, Nigeria, Sierra Leone | RCT-PC | 18 months  70 years  (incl. PLWH) | 612 | Tolerability, immunogenicity | CEPI, PHAC, IAVI | Ongoing (estimated completion 04/2027) | NCT05868733 / PACTR202210840719552 [(41,42)](https://paperpile.com/c/rCl9nY/Moy8Q+TRm90) | Lower Dose (2×10^6^ pfu)  Higher Dose (1×10^7^ pfu) |
| Recombinant viral vector | rVSVΔG-LASV-GPC | 1 | 2021 | USA, Liberia | RCT-PC | 18–50 years | 110 | Safety, tolerability | CEPI, IAVI | Completed – results partially published (Malkin et al.) (03/2024) | NCT04794218 / PACTR202106625781067. [(37)](https://paperpile.com/c/rCl9nY/waoJs) | 2 × 10^^4^ pfu –1 or 2 doses  2 × 10^^5^ pfu - 1 or 2 doses  2 × 10^^6^ pfu - 1 or 2 doses  2 × 10^^7^ pfu - 1 or 2 doses |
| Recombinant viral vector | EBS-LASV (Emergent BioSolutions) | 1 | 2021 | Ghana | RCT-PC | 18–50 years | 108 | Safety, immunogenicity | CEPI (platform support) | Discontinued | PACTR202108781239363 [(43)](https://paperpile.com/c/rCl9nY/Od6ZF) | 10^^5^ - 2 doses  10^^6^ - 2 doses  10^^7^ - 2 doses  1-29 days |
| Recombinant viral vector | MV-LASV (Themis / BioNTech) | 1 | 2019 | Belgium | RCT-PC | 18–55 years | 60 | Safety, immunogenicity | Merck (via Themis) | Discontinued – development halted (Tchismarov et al) | NCT04055454 [(36)](https://paperpile.com/c/rCl9nY/qelrD) | 2 × 10^^4^ × 2 doses  1 × 10 ^^5^ - 2 doses |
| DNA | INO-4500 | 1 | 2021 | Ghana | RCT-PC | 18–50 years | 220 | Safety, immunogenicity | CEPI, Inovio | Discontinued – no Phase 2 planned | NCT04093076 [(44)](https://paperpile.com/c/rCl9nY/Tzxn4) | 1mg-2mg. One dose |
| DNA | INO-4500 | 1 | 2019 | USA | RCT-PC | 18–50 years | 60 | Tolerability, immunogenicity | Inovio | Discontinued | NCT03805984 [(44)](https://paperpile.com/c/rCl9nY/Tzxn4) | 1mg-2mg. One doses |
| Inactivated viral vector | LASSARAB | 1 | 2025 | USA | RCT-PC | 18–50 years | 55 | Safety, immunogenicity | Maryland University | Ongoing | NCT06546709  [(45)](https://paperpile.com/c/rCl9nY/9ilr4)  Ongoing, not recruiting | One or two doses |
| Recombinant viral vector | ChAdOx1 LassaJ | 1 | 2024 | UK | RCT-PC | 18-55 years | 31 | Safety, immunogenicity | CEPI, Oxford Vaccine Group | Ongoing  Still not recruiting | [(40)](https://paperpile.com/c/rCl9nY/kDvwl) | 5 x 10^^10^. Two doses |

# **S1 Table 5. Consolidated summary of adverse events by candidate and time window**

This table summarizes solicited local/systemic adverse events (0–30 days and 31–180 days post‑vaccination), stratified by candidate (rVSVΔG‑LASV‑GPC; MV‑LASV). Event rates per 100 participants with 95% confidence intervals are presented for arthralgia, diarrhea, fever, and myalgia. See S1 Fig A–D for forest plots and methods for synthesis details.

| **Candidate** | **Time window** | **Arthralgia (per 100)** | **Diarrhea (per 100)** | **Fever (per 100)** | **Myalgia (per 100)** |
| --- | --- | --- | --- | --- | --- |
| rVSVΔG‑LASV‑GPC | 0–30 days | 32.02 (12.48–54.82) | 11.85 (1.44–27.60) | 31.49 (10.73–56.24) | 56.22 (20.78–88.82) |
| MV‑LASV | up to Day 56 | 2.71 (0.00–16.45) | 18.47 (8.31–31.13) | 4.16 (0–12.60) | 24.99 (13.38–38.57) |
