# Supplementary material for: Efficacy, safety, and immunogenicity of Lassa fever vaccines: A living systematic review and landscape analysis of vaccine candidates
Source: PLoS One. 2025 Dec 17;20(12):e0338128. doi: 10.1371/journal.pone.0338128 (PMC12711090; doi:10.1371/journal.pone.0338128)
Supplement: Attachment — Submitted filename: Response_to_Reviewers_Lassa (1).docx [file pone.0338128.s006.docx]

Response to Academic Editor and Reviewers

Manuscript ID: PONE-D-25-48423
Title: Efficacy, safety, and immunogenicity of Lassa fever vaccines: a living systematic review and landscape analysis of vaccine candidates
Due date for resubmission: November 21, 2025 11:59 PM (America/Argentina/Buenos_Aires)

We thank the Academic Editor and reviewers for their constructive comments. Below we provide a point-by-point response. Reviewer comments are in italics; our responses follow, with page/line references to the revised manuscript.

# Editor: Additional requirements and technical points

**PRISMA for Abstracts checklist:** We have added a PRISMA for Abstracts checklist as a separate file and ensured the abstract complies with itemized reporting.

**Explicit SR objective:** Added at the end of the Introduction (Objective paragraph).

**Methods transparency:** Methods now explicitly report databases searched, inclusion/exclusion criteria, study selection, data items, synthesis/heterogeneity handling, missing data, and ethics for animal studies.

**Outcomes and variables:** Data items and outcomes are now explicitly listed, including operational definition of seroconversion.

**Figure captions:** We have ensured separate figure legends for all figures in the manuscript.

# Reviewer #1

*Comment: Provide bibliographic references for all 51 included studies.*

Response: Done. We cite each included study in Tables and in the References

*Comment: Title reflects reactogenicity/tolerability and order of outcomes*

Response: We retained the main title per PLOS ONE guidance but revised the abstract/results to present safety before immunogenicity and clarified tolerability/reactogenicity.

# Reviewer #2

*Comment: Abstract length and focus*

Response: Abstract reduced to ≤300 words with emphasis on quantitative results and cautious conclusions.

*Comment: Introduction updates and justification for including preclinical data*

Response: We updated epidemiology references and added a brief justification for combining clinical and preclinical evidence and guidance on interpretation.

*Comment: Methods—criteria for preclinical evidence, handling unpublished/abstract-only data, heterogeneity and missing data, animal ethics*

Response: All topics clarified as requested; GRADE considerations for lower‑certainty abstract‑only data are described.

*Comment: Results—definition of seroconversion; AE summary table; moving long landscape tables to Supplement; separate mapping vs synthesis*

Response: Seroconversion definition added; we created a consolidated AE summary table in the Supplement; detailed landscape tables moved to Supplement with highlights in main text; mapping vs synthesis distinctions emphasized. We inserted abridged tables instead in the main paper.

*Comment: Discussion—cautious interpretation, equity/ethics, correlates of protection, and LSR practicalities*

Response: We tempered statements, expanded equity/ethics discussion, highlighted uncertainty around correlates, and reflected on strengths/challenges of an LSR.

*Comment: Archiving of supplementary materials with DOIs*

Response: We are prepared to archive the Supplement and dashboard exports in Zenodo/OSF with DOIs upon acceptance; we added this note to the Data Availability statement.

*Comment: Language and style*

Response: We streamlined the abstract and discussion to reduce redundancy and improve readability.

*Comment: References—duplicates/URLs*

Response: We screened for duplicates and verified URLs where applicable.

On behalf of all co-authors,
Ariel Bardach
